# Facile Fabrication of Superhydrophobic Copper-Foam and Electrospinning Polystyrene Fiber for Combinational Oil–Water Separation

**DOI:** 10.3390/polym11010097

**Published:** 2019-01-08

**Authors:** Yu-Ping Zhang, Jing-Hua Yang, Ling-Li Li, Cheng-Xing Cui, Ying Li, Shan-Qin Liu, Xiao-Mao Zhou, Ling-Bo Qu

**Affiliations:** 1 Henan Institute of Science and Technology, Xinxiang 453000, China; lilacyjh@163.com (J.-H.Y.); linglili_linglili@163.com (L.-L.L.); chengxingcui@hist.edu.cn (C.-X.C.); hnliy0412@126.com (Y.L.); lsq.0508@163.com (S.-Q.L.); 2 Hunan Academy of Agricultural Sciences, Changsha 410128, China; 3 College of Chemistry and Molecular Engineering, Zhengzhou University, Henan 450001, China; qulingbo@zzu.edu.cn

**Keywords:** copper foam, superhydrophobicity, polystyrene, electrospinning, oil–water separation

## Abstract

Membrane-based metal substrates with special surface wettability have been applied widely for oil/water separation. In this work, a series of copper foams with superhydrophobicity and superoleophilicity were chemically etched using 10 mg mL^−1^ FeCl_3_/HCl solution with consequent ultrasonication, followed by the subsequent modification of four sulfhydryl compounds. A water contact angle of 158° and a sliding angle lower than 5° were achieved for the copper foam modified using 10 mM *n*-octadecanethiol solution in ethanol. In addition, the interaction mechanism was initially investigated, indicating the coordination between copper atoms with vacant orbital and sulfur atoms with lone pair electrons. In addition, the polymeric fibers were electrospun through the dissolution of polystyrene in a good solvent of chlorobenzene, and a nonsolvent of dimethyl sulfoxide. Oil absorption and collection over the water surface were carried out by the miniature boat made out of copper foam, a string bag of as-spun PS fibers with high oil absorption capacity, or the porous boat embedded with the as-spun fibers, respectively. The findings might provide a simple and practical combinational method for the solution of oil spill.

## 1. Introduction

Constructing an extremely nonwetting surface onto different substrates with the water contact angles (WCAs) more than 150° is a great challenge for its tremendous application in areas, such as self-cleaning [1] and anti-icing surfaces [2,3] drag reduction [4], and oil-water separation [5]. Recently, membrane-based metal meshes with special surface wettability have attracted a significant amount of attention for the treatment of increasing industrial oily waste water, as well as frequent oil spill accidents [6,7]. Due to its excellent thermal and electrical properties, copper, an essential engineering material, is widely used in many fields, including the chemical industry, electronic industry, and computer technology [8,9]. Also, copper foil, meshes, or porous substrates are often widely chosen as oil-water separation materials by researchers because of its availability, low cost, and superior mechanic properties and high oil-water separation efficiency [10]. Oil-water separation could be used in many industrial fields, especially in a crude-oil-treating facility, which could be found from some excellent industrial reports [11,12]. In the past several years, a lot of methods were reported for processing copper substrates with a superhydrophobic surface for oil–water separation. These methods were generally carried out by electrodeposition [13], chemical etching [14], ultrasound irradiation [15], chemical vapor deposition (CVD) [16], and so on.

The common procedures to fabricate the superhydrophobic surface on Cu substrates include two important steps: One is how to achieve a rough face on the micro- and nano-scales, the other is how to modify a firm film or membrane with a low-surface energy effectively. Cu surfaces were easily achieved by simple acid or base etching, replacement deposition, and electrodeposition [11,12,13,14,15,16]. In general, micro- and nano-scale structures on the hydrophobic surfaces of these copper substrates were usually involved in many types of morphology and different modified compounds with a low surface energy. Shirtcliffe et al. developed a method to construct a nanoribbons’ surface with a size of 1 µm × 100 nm × 6 nm, which was coated with a commercial fabric waterproofing agent sequentially. The uniform superhydrophobic layer on the inside of round copper tubes of a millimetric internal radius could enhance flow rates and reduced drag for both water and 50% *w*/*w* water-glycerol mixtures [17]. Chen et al. used a one-step solution-immersion process to prepare a compact and uniform superhydrophobic film, the leaf-like and compact submicroscopic structure was maintained in ethanol solutions of *n*-dodecanethiol and tetradecanoic acid. Meanwhile, the results of theoretical calculations showed the bonding style was two Cu atoms combined with one S atom [18]. Chaudhary et al. prepared a superhydrophobic CuO/Cu(OH)_2_ coating on the copper sheet surface with microflowers and nanoneedle arrays by a simple solution-immersion in a mixture of 2.0 M sodium hydroxide and 0.15 M ammonium persulfate at room temperature, without using a low surface energy material [19]. A similar etching method was developed with the H_2_O_2_/HCl etchants, followed by the modification of stearic acid. The as-prepared surface was composed of many convex polyhedral protrusions with diameters ranging from 0.5 to 2 μm and many cavities included sizes of hundreds of nanometers on these protrusions [20]. Cheng et al. fabricated a mesh film with pine-needle-like structures, followed by the modification of the mixed thiol (containing both alkyl and carboxylic acid groups). The formed Cu(OH)_2_ on the surface possessed a diameter of 80−150 nm and a length of about 8 μm [21]. Other typical structures onto the copper substrates also include the non-flaking bicrystalline CuO nanowires [22]. The pine-cone-like hierarchical micro-nanostructure [23], the surface distributed grooves and ridges in micrometer scale and many nano-wrinkles [24], and the unique nanoworm-like structure [25] were mainly modified by fluorosilane previously. Also, some other examples involve rodlike nanostructures of Cu(OH)_2_ [26,27], highly dense ordered Cu_2_O nanorods [28], flower-like [29], leaves-like, and wormlike micro-/nano-scale structures [30,31], and these hydrophobic surfaces were finally modified by *n*-alkanethiolates [32]. To some extent, the techniques described above were efficient for separating mixtures of water and oil, however, there are some disadvantages that need to be overcome, such as time-consuming and complicated fabrication, special equipment, and expensive materials. Especially, very few reports have involved the interaction mechanism on the superhydrophobic surface.

In this article, a simple solution-immersion and simultaneous ultrasonication with subsequent modification of four typical sulfhydryl compounds were developed to fabricate the superhydrophobic surface. It was composed of multiscale hierarchical irregular structures, including pure Cu phases, micro-/nano-size particles, and organic films. In addition, the modification mechanism of sulfhydryl compounds onto the surfaces of Cu foams was initially explained, suggesting the main coordination between copper atoms with vacant orbital and sulfur atoms with lone pair electrons. Moreover, the miniature boat made out of Cu foam and loaded with as-spun polystyrene (PS) fibers provided a good solution for the oil cleanup on the water surface. When our miniature oil boat floats on the surface of oil-water mixtures, oil could penetrate through the walls of Cu foam and then diffuse into the as-spun fibers embedded in a string bag rapidly, whereas water was completely repelled. Additionally, the adsorbed oil in the fibers were quickly released by squeezing, thus achieving an efficient way to collect the absorbed oil.

## 2. Experimental

### 2.1. Materials

Copper foams (purity ≥ 99.999%, pore size 0.1 mm) were purchased from Suzhou Taili Metallic Foam Factory. *n*-ethanethiol (EE), *n*-dodecanethiol (DE), *n*-hexadecanethiol (HDE), and *n*-octadecanethiol (ODE) were purchased from Tianjin Kermel Chemical Reagent Co. (Tianjin, China). Ferric chloride, ethanol, acetone, phenixin (CCl_4_), *n*-hexane, and chloroform (CHCl_3_) were obtained from Jiangsu Yonghua Fine Chemistry Co. (Changshu, China). Oil red (Sudan III), methylene blue, allura red, NaOH, HCl, NaCl, ethanol, and methanol were obtained from Tianjin Fine Chemical Co. (Tianjin, China). Gasoline engine oil, rapeseed oil, castor oil, coal tar, and peanut oil were purchased from a local market. Polystyrene (PS, average Mw~192,000), chlorobenzene (CB), and dimethyl sulfoxide (DMSO) were purchased from Sinopharm Chemical Reagent Co., Ltd. (Shanghai, China). The current adopted solvents, such as chlorobenzene, chloroform and DMSO, are typical pollutants, which could cause environmental impacts for large scale application. These solvents could be recycled with green technology, such as membranes [33,34].

### 2.2. Fabrication of Superhydrophobic Cu Foam

**Scheme 1:**
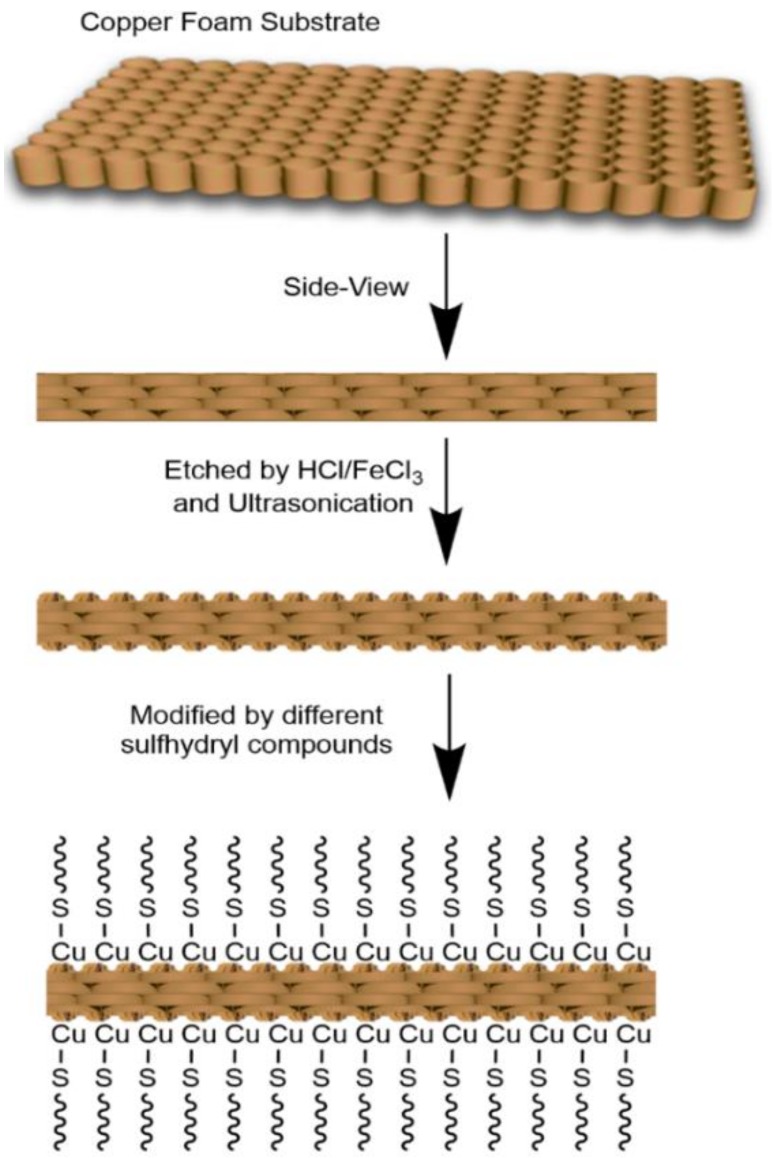
Typical scheme for the fabrication process of the superhydrophobic copper foams.

The superhydrophobic Cu foams were prepared through a similar method as already reported [31]. The procedure of fabricating the superhydrophobic surface onto Cu foam is briefly illustrated in Scheme 1, which includes the chemical etching in FeCl_3_/HCl mixed solutions to create rough structures and subsequent modification with four sulfhydryl compounds to reduce the surface energies. Briefly, a piece of Cu foam (10 × 10 × 1.5 mm, 30 × 30 × 1.5 mm) was firstly ultrasonic rinsed with acetone, ethanol, and deionized water for several times sequentially, and then desiccated with N_2_. FeCl_3_ was dissolved in 0.1 M HCl in order to keep the acidic condition during the reaction. Then, a piece of the cleaned Cu foam was immersed in FeCl_3_ solution in the concentration range of 2.5–40 mg/mL for 5–25 min with consequent ultrasonication, followed by water washing, and drying. After the growth of nanostructures, the substrates were covered with a rough layer and modified with each thiol solution for 5–25 min, respectively. The concentration for four thiol compounds was selected in the range of 1–20 mmol L^−1^ in ethanol. At last, these Cu foams were rinsed with abundant ethanol and desiccated with N_2_.

### 2.3. Fabrication of PS Fiber

The electrospinning fibers were prepared using a single-capillary spinneret with a feed rate of 0.8 mL h^−1^. PS was mixed in solution of chlorobenzene (CB), and dimethyl sulfoxide (DMSO) with different ratios, followed by magnetic stirring at 50 °C for 12 h. All the experiments were carried out at room temperature, a humidity of 50%–70%, and an applied voltage of 14 kV.

### 2.4. Wettability Measurement and Characterization

The water contact angles (WCAs) at room temperature (about 4.5 μL of distilled water droplet was used for the measurements) were measured according to the sessile-drop method using a contact angle analyzer (SPCA, Beijing HaKe Test Instrument Factory, Beijing, China). The drop image was stored by the video camera, and an image analysis system was used to calculate the left and right angles from the shape of the drop. The droplet size of the fluid was about 5–50 μL. For the water sliding angles (WSAs) measurement, the substrate was placed on a custom designed stage measured with a protractor and a water droplet of 5–50 μL was used. All WCA and WSA values were averaged over three different measurements on each sample.

A FEI QUANTA 200 scanning electron microscope (Philips-FEI Corporation, Netherlands) was used to study the surface morphology of the foam copper and as-spun fiber. Before SEM observation, all samples were fixed on aluminum stubs and coated with gold. The surface composition was characterized by X ray diffraction (DX2700B-XRD, Dan Dong Hao Yuan Instrument Co., Ltd., Dandong, China). The impacting processes of water and oil onto tested Cu foams were monitored using a high-speed camera (video camera AG-HMC153MC, Panasonic, Inc., Osaka, Japan), for macroscopic and microcosmic observations, respectively.

### 2.5. Stability in Rigid Condition

In the stability evaluation, the as-prepared Cu foams were dripped a drop of 0.1 M HCl, 0.1 M NaOH, and 3.5% NaCl solutions, respectively. The immersion time was about 5 min, 10 min, and 10 h, respectively. After that, the samples were washed with deionized water and dried by nitrogen.

### 2.6. Oil/Water Separation and Oil Absorption of Superhydrophobic Cu Foam

The typical Cu foams modified by ODE were used for the oil/water separation using five types of oil with different viscosity and three typical organic solvents, respectively. The separated oil/organic solvent and water were collected with beakers to measure the separation efficiency.

## 3. Results and Discussion

### 3.1. Chemical Etching and Post-Modification

The wet etching process was based on the oxidation–reduction reactions between the Fe^3+^ and Cu, which is initiative and rapid, but enhanced by ultrasonic irradiation. Strong acid atmosphere (0.1 M HCl) could provide a stable and favorable oxidizing environment, so that the possible oxidation layers (CuO or Cu_2_O) onto the surfaces of the Cu foam were easily dissolved and hydrolyzation of Fe^3+^ was minimized effectively. Herein, ultrasonication increased the etching velocity through cavitation (bubble formation, growth, and collapse) and heating. When the microscopic cavitation bubbles collapsed near the surfaces of the Cu foam, they generated powerful shock waves and microjets that caused effective stirring/mixing of the acid and FeCl_3_ solutions. The numerous bubbles evolving at different locations on the pure copper surface that function as static and dynamic templates, so that many square nanoparticles were formed unevenly. The solution color was gradually changed from yellow to green, indicating Fe^3+^ ions were reduced to Fe^2+^ ions, the color changes among the pores of the Cu foam were especially obvious if the etched foam was only rinsed with deionized water for several times (see Figure S1). Green color was observed among the pores of the Cu foam after several hours, indicating the existence of residual Cu and Fe ions onto the surfaces. In contrast, no green color was found among the pores of the copper foam after the etched surface was washed by deionized water several times and desiccated with N_2_ fully. That is to say, the attached ions could be removed from the etched surface thoroughly via the combination of washing and desiccating methods. The post-modification of sulfhydryl compounds was carried out quickly by immersing the Cu foams into the ethanol solutions of EE, DE, HDE, and ODE, respectively. In Video S1 (Supporting Information), many water droplets were carefully added onto the fabricated foams by a micropipette of 20 μL. The blue and red waterdrops were dyed by methylene blue and allura red, respectively. These 5–20 μL waterdrops with less adhesive force onto the smooth surfaces could be removed easily by the tip of a micropipette. Four capital letters of HIST were written on the foam surfaces, with H, I, S, and T standing for the Cu foams modified by EE, DE, HDE, and ODE, respectively (see Scheme S1). It should be noted that to attach these waterdrops regularly was very difficult due to their non-sticky behavior, as they could roll around easily onto the superhydrophobic surfaces. All Cu foams, but the EE foam, had good superhydrophobicity, indicating the alkyl chains (CH_3_(CH_2_)_4_COO–, CH_3_(CH_2_)_10_COO–, CH_3_(CH_2_)_14_COO–, and CH_3_(CH_2_)_16_COO–) were chemically grafted at the etched surfaces of their respective Cu foams.

### 3.2. Structure and Morphology

**Figure 1:**
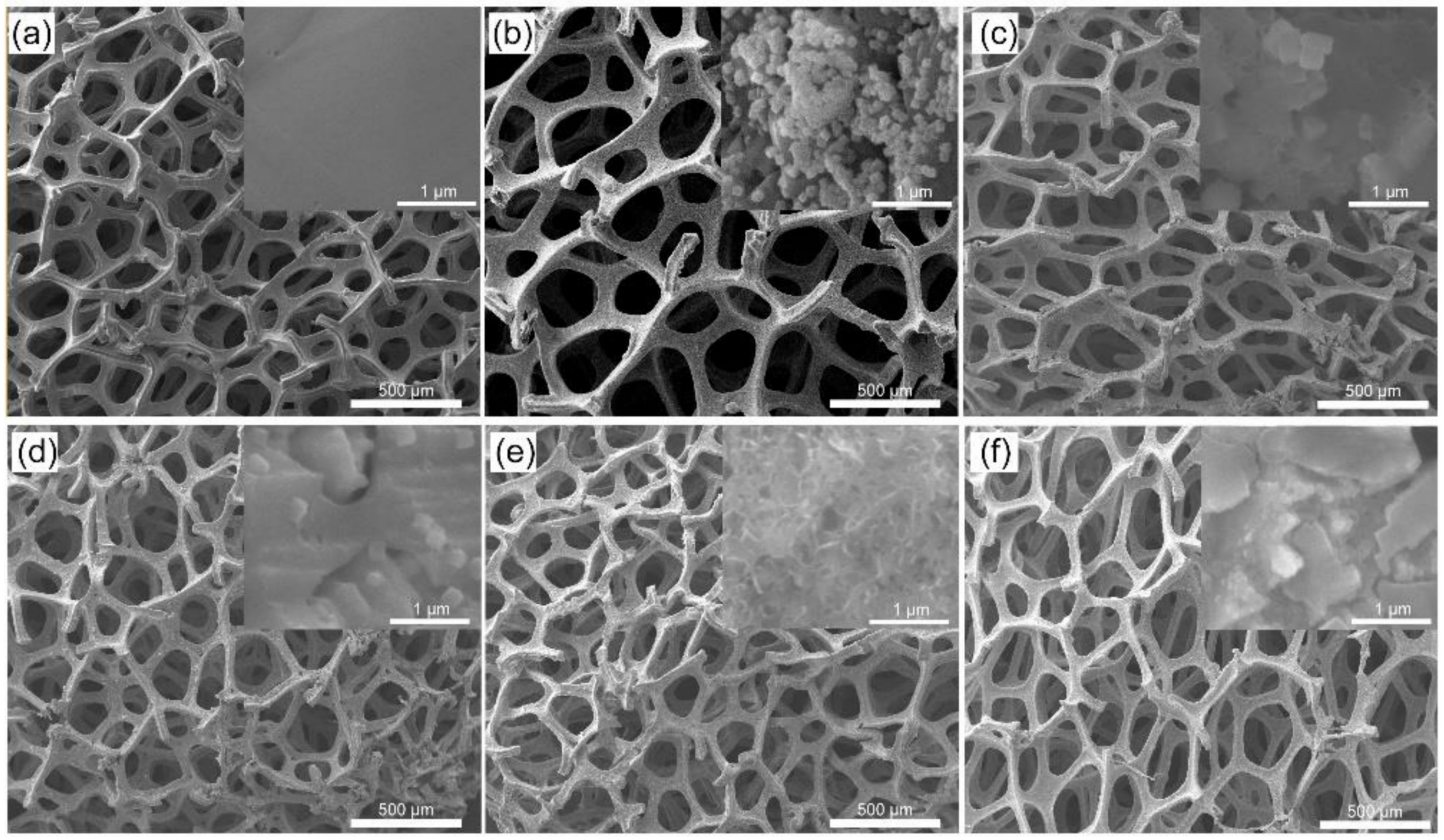

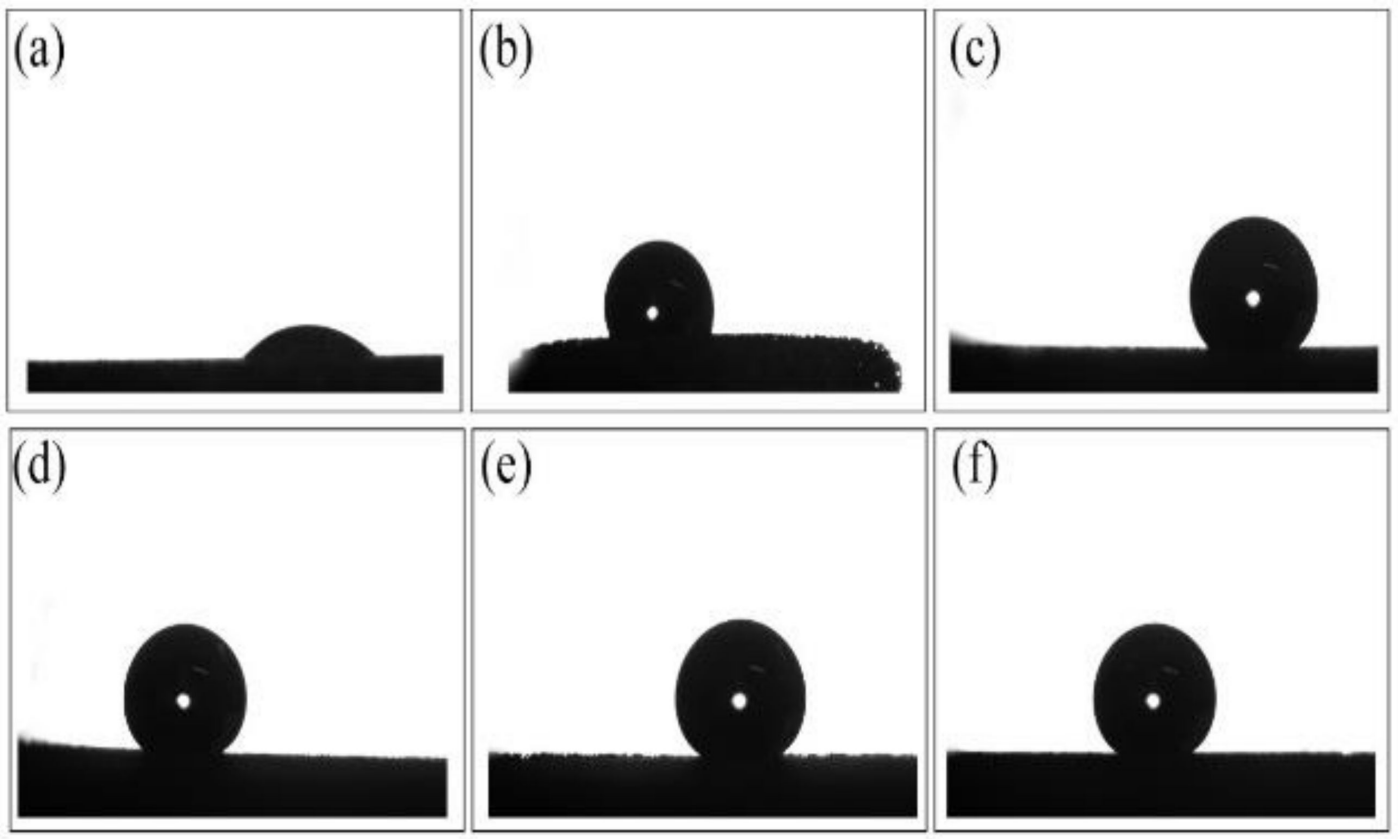
**Upper**: SEM characterization of the foam coppers (scale bar = 500 μm). Numbers (**a**–**f**) stand for the blank copper foam, etched copper foam, copper foams modified using 15 mL EE (*n*-ethanethiol), DE (*n*-dodecanethiol), HDE (*n*-hexadecanethiol), and ODE (*n*-octadecanethiol) with an identical concentration of 1 mM, respectively; the insets (**a**–**e**) show the nanoscale patterns. The insets with a scale bar of 1 μm show the enlarged view of the surface. **Below**: The WCAs of the Cu foams determined.

Figure 1 (upper) shows the SEM images of the Cu surface with different magnifications for the blank Cu foam, the etched Cu, and the modified Cu foams using *n*-EE, *n*-DE, *n*-HDE, and *n*-ODE, respectively. The commercial copper foam possessed a pore size in the range of 100–500 μm and a skeleton width in the range of 20–30 μm. After being etched and/or modified by sulfhydryl compounds, no apparent changes were found for the pore sizes and the skeleton widths. However, as shown in the insets, with 1 μm scaleplate, the blank Cu foam was observed with a very smooth surface (Figure 1a), but many cube particles were found for all other Cu foam surfaces on the micro-scale roughness surfaces (Figure 1b–f). Such a dual-scale hierarchical structure favored the superhydrophobicity of the Cu surface. In general, Cu foam was polycrystalline and its grains were distributed randomly. The FeCl_3_/HCl etching behavior preferentially occurred on the randomly distributed facets or grain boundaries, so the irregular surface with micro/nano structure was created.

The WCAs of the resulted Cu foams were measured in Figure 1 (below). The changes of wetting abilities were attributed to the micro/submicrometer structures onto the Cu surfaces and the modified sulfhydryl compounds with low surface energy [35,36,37,38]. The wetting performance decreased obviously after the blank copper foam that was etched with the WCAs increased from 40° to 108.3°. In addition, the longer the alkyl chains for the modified sulfhydryl compound, the stronger the hydrophobic ability. The resulted copper foams modified with the alkyl chain of C_6_, C_12_, C_16_, and C_18_, corresponded to their WCAs of 144°, 155°, 157°, and 158°, respectively. The greatest water-repellency was achieved for the substrate modified by ODE, with a WSA less than 5° (see Videos S2–S4).

**Figure 2:**
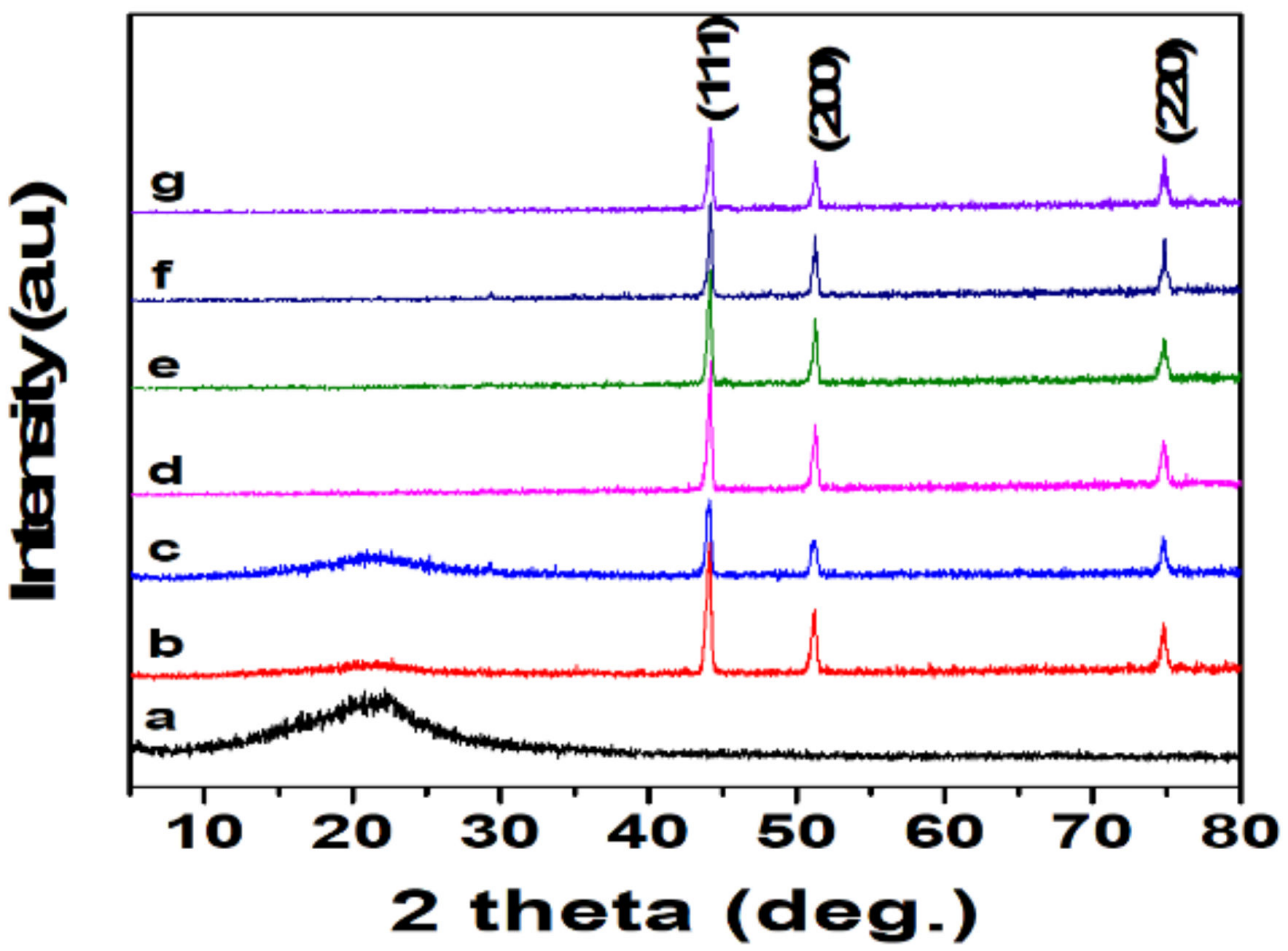
XRD patterns of (**a**) glass plate and double faced adhesive tape; (**b**) Cu foam; (**c**) Cu foam etched by FeCl_3_; (**d**–**g**) etched Cu foams modified by EE, DE, HDE, and ODE, respectively.

The XRD spectra of the surface of bare Cu foam and the as-prepared surfaces after being etched and/or modified by four sulfhydryl compounds are shown in Figure 2. From the pattern, it was clear that all Cu substrates except the glass plate tied with double faced adhesive tape had three typical peaks at 43.5°, 50.6°, and 74.3° for the indices (111), (200), and (220), respectively, suggesting all Cu foams contain a cubic copper phase [23,39]. If the Cu foam surfaces existed as oxides, the spectra should include their typical peaks of (110), (111), (200), (211), and (220) for the Cu_2_O phases, and of (110), (200), (220), (311), and (222) for the CuO phases, respectively [21,23,40,41]. From the results that XRD patterns agreed well with the reported data of the Cu element apparently, it could be believed that the Cu foams before and after being etched and/or modified were mainly comprised of pure Cu material devoid of any metal oxides of Cu_2_O and CuO in the crystallized part of pure material.

It is reasonably believed that the main reaction includes the initial redox between the Cu atom and FeCl_3_ enhanced by ultrosound irradiation. The important evidence was that almost all of Cu and Fe ions were removed from the surface after being washed by deionized water and desiccated with N_2_ fully. Another reaction was that sulfhydryl molecules chemisorbed on Cu by the dissociative adsorption of a S−H bond to yield a copper thiolate [41,42,43,44]. The hydrogens in the S−H bond were first chemically adsorbed to Cu and thus H_2_ is formed. The relative reactions can be described as follows:

Cu + 2 FeCl_3_ → CuCl_2_ (green) + 2 FeCl_2_ (green)

Cu + C_18_H_37_SH → C_18_H_37_SCu + ½ H_2_

In fact, as shown in Scheme S1, the oxidation–reduction reaction and the interaction with the help of the coordination bond together constitute the main body of the aforementioned chemical synthesis mechanism.

### 3.3. Optimization of the Etching Process and Modification

**Figure 3:**
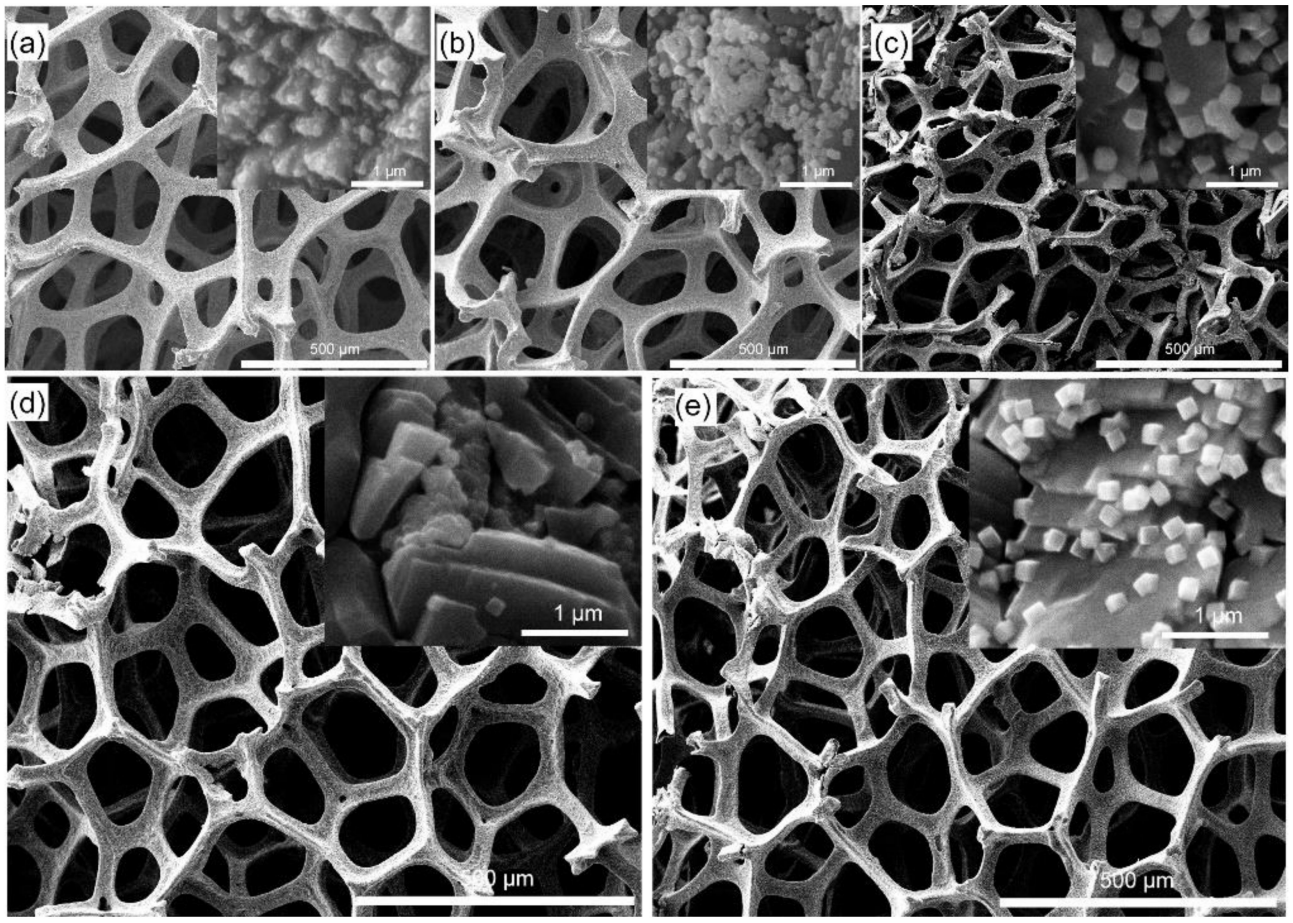
SEM characterization of Cu foam immersed into a mixture aqueous solution of FeCl3 (0.01 g mL^−1^) and HCl (0.1 M) at room temperature (scale bar = 500 μm). Numbers (**a**–**e**) stand for the immersion time for 5, 10, 15, 20, and 25 min, respectively. The insets (**a**–**e**) show the nanoscale patterns on the microscale roughness surface. The insets with a scale bar of 1 μm show the enlarged view of the surface.

The immersion time and the concentration of FeCl_3_ and HCl played a vital role during the preparation process. The surface morphology of the Cu foams after immersion and ultrosonication in the acid aqueous solution of FeCl_3_ for different times was characterized by SEM in Figure 3. It could be seen that the immersion time of 10 min was enough to create many nano-size particles onto the surfaces with the help of the chemical reaction and ultrasonication. Furthermore, the mass of Cu components decreased with the progressive oxidation-reduction reaction. The Cu amount removed from the original substrate after 10 min immersion was decided by the amount of Fe^3+^ in solution.

When the concentration of FeCl_3_ increased from 2.5 mg mL^−1^ to 40 mg mL^−1^, the final mass of each piece of Cu foam (10 × 10 × 1.5 mm) gradually decreased, as shown in Table S1 and Figure S2 (Supporting Information). The whole Cu foam was depleted fully when 20 mL to 40 mg mL^−1^ FeCl_3_ solution was added for 10 min immersion due to a strong oxidation-reduction reaction. It should be noted that a different oil absorption amount was changed in the range of 3.8–5.3 times itself of the amount of the copper foams etched with Fe^3+^ with different concentrations and modified with 1 mM ODE identically. The greatest absorption ratio of gasoline engine oil was obtained for the Cu foams using 10 mg mL^−1^ FeCl_3_ solution.

Five pieces of Cu foams etched by 10 mg mL^−1^ FeCl_3_ solution were immersed in ODE ethanol solution for the optimization, and the concentration was varied in the range of 1–20 mM with a constant immersion time of 15 min. The morphology and WCAs were characterized and determined in Figure S3 (Supporting Information). It should be noted that the formed films onto the rough surface were very thin so that no obvious differences of morphology were recognized, but the original cube nanoparticles. Similar values of WCAs showed that the concentration effect of ODE on the wetting behavior could be ignored in the selected range. Moreover, the immersion time was changed in the range of 5–25 min with a constant ODE concentration of 10 mM. After the modified porous substrates were rinsed with ethanol and dried, they were selected for the determination of superhydrophobic properties. As shown in Table 1, the WCAs were achieved in the range of (150 ± 1.5)°–(158.3 ± 1.5)° with a WSA less than 5° and the largest absorbing amount of gasoline engine oil was obtained using 10 mM ODE.

According to the above experimental results, the optimal conditions to prepare the Cu foams are described as follow: 10 mg mL^−1^ FeCl_3_ in 0.1 M HCl was used for the etching process about 10 min with consequent ultrosonication. After the etched foam was rinsed by water and dried by N_2_, 5 mL 10 mM *n*-ODE in ethanol solution was then applied for the modification about 10 min, respectively.

### 3.4. Water Rebounding and Oil Penetrating Experiments

**Figure 4:**
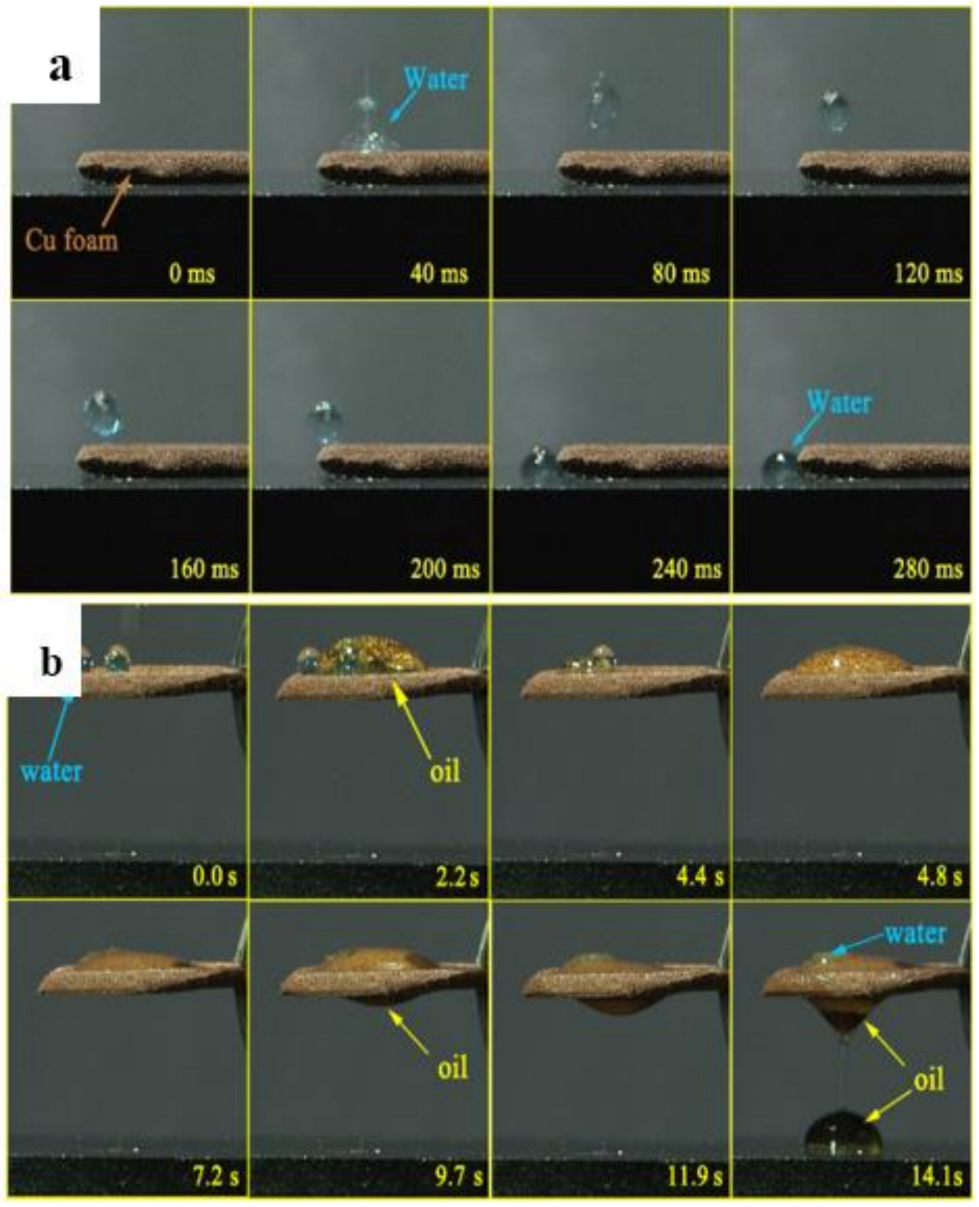
A series of snapshots showing a droplet of water bouncing and oil penetrating on the prepared copper foam. (**a**) Bouncing process of a water droplet. (**b**) Penetrating process of an oil droplet. Snapshots of a water droplet impacting a superhydrophobic copper foam surface. The snapshots were obtained using a high-speed camera. The water droplet and gasline engine oil continually falls freely from a height of H = 6 cm and 8 cm, respectively. The volumes of each water droplet and gasline engine oil is 50 µL, respectively.

To examine liquid wetting behaviors of as-prepared Cu foam more intuitively, a high-speed camera system was used to record the gravity-driven oil–water separation process in Figure 4. The processes of water droplet bouncing and oil penetrating were videotaped, respectively. The impacting water droplet viewed from the side (upper of Figure 4) spread to the surface, retracted, and then lifted off within 240 ms, until it rolled away from the surface. Little contact time between water droplets and the as-prepared interface proved that Cu foam had a fine ability to resist water. Due to the capillarity effect and van der Waals attractions, the gasoline engine oil had priority, compared with water (dyed with methylene blue), to pass through the as-prepared Cu foam. By simple initial immersion in oil solution, the foam surface could easily adsorb oil molecules due to its sticky force, thus this made oil pass through easily within 14.1 s. In the whole process, water droplets were observed to remain on the foam surface until most of the oil passed through the foam. Furthermore, when a waterdrop continuously hit the foam surface, it first flocked together to form a big water droplet, vibrated on the surface, and finally rolled away from the surface without being able to penetrate the surface. One possible explanation might be that the synergistic effect of both internal physical structures. Another key factor might come from the external chemical compositions of the current system. At the molecular level, Cu networks were composed of hydrophobic alkyl chains (CH_3_(CH_2_)_16_COO–), ensuring the strong resistance ability for water molecules.

### 3.5. Oil-Water Separation and Oil Absorption

**Figure 5:**
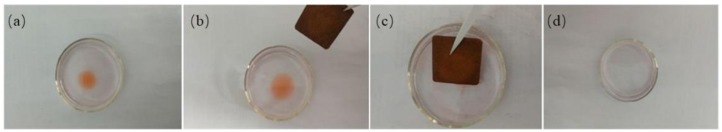
Optical images of copper foam for the cleanup of hexane on the water surface (**a**–**d**).

In an attempt to investigate the uptake capacities of the as-prepared Cu foam, the following experiment was carried out as shown in Figure 5. Many dyed *n*-hexane (Sudan, red) droplets were dropped onto the middle of the water surface in a watch glass (Figure 5a), and then a piece of fabricated copper foam with a size of 30 mm × 30 mm was held to approach the organic droplets (Figure 5b). The dyed *n*-hexane (Sudan, red) was quickly absorbed by the superhydrophobic copper foam for about 10 s (Figure 5c,d). The organic droplets were taken up quickly, indicating the difference of surface energy between water and *n*-hexane (see Video S5, Supporting Information).

**Figure 6:**
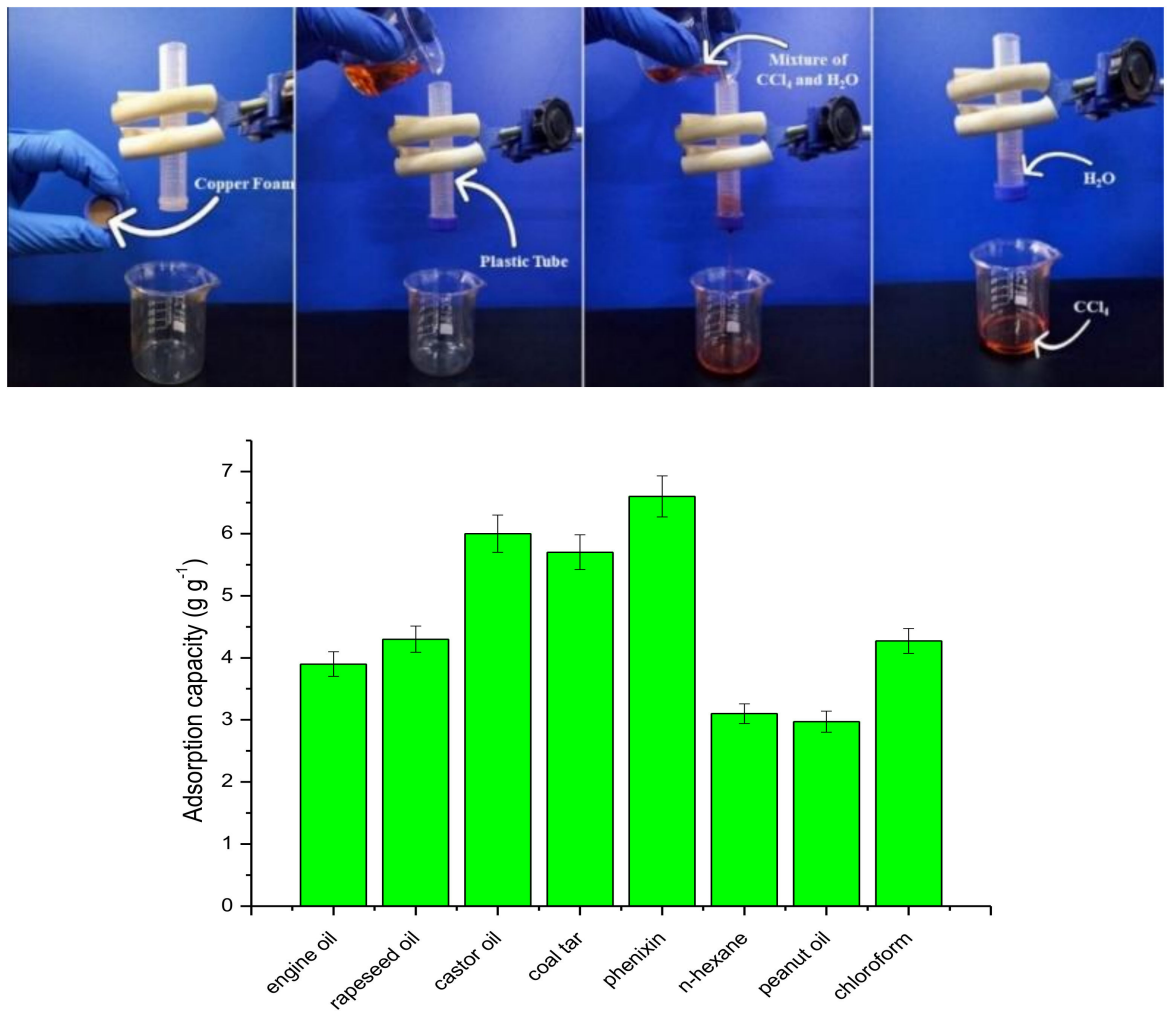
A photograph of the oil-water separation procedure (**upper**) and absorption capacity for different oils, such as gasoline engine oil, rapeseed oil, castor oil, coal tar, phenixin, *n*-hexane, peanut oil, and chloroform (**below**).

Moreover, utilizing the superhydrophobic and superoleophilic copper foam, an oil/water separation apparatus was constructed as shown in Figure 6 (upper) and Video S6 (Supporting Information). The flexible copper foam was immobilized in the rubber plug, where the vertical plastic tube was passed through. After the rubber plug was screwed on the plastic tube tightly, the mixture of 5 mL CCl_4_ (red) and 5 mL water was poured in the plastic tube, thus CCl_4_ quickly permeated through the foam, owing to its superoleophilic nature, and dropped into the beaker below, while water was retained above the foam due to its superhydrophobic nature. Following this procedure, eight types of oil and organic solvents, including gasoline engine oil, rapeseed oil, castor oil, coal tar, peanut oil, *n*-hexane, peanut oil, and chloroform, were successfully separated by using this simple filtration set-up.

After the separation process was carried out, the oil was collected and weighed, respectively. The oil separation efficiency (*E*s) was calculated by the equation, *E*s = *V*/*V*_0_, where *V*_0_ is the volume of the oil in the oil−water mixture, and *V* is the volume of the oil, which is collected in the end. Taking CCl_4_ as an example, the separation efficiency of the copper foam was above 97% for the first cycle. Even after 10 cycles, the as-prepared metallic foam still performed well with a separation efficiency up to 95% and no obvious decrease of its absorption capacity was found, exhibiting its long-term repeatability (see Figure S4). Although the Cu foams could only absorb the selected five oils and three organic solvents 3.0–6.6 times compared to its own weight (Figure 6, below), it was still a good choice for oil/water separation as a porous superhydrophobic substrate. In addition, the durability and stability of the as-prepared copper foams treated with ODE were investigated by dipping in 0.1 M HCl, 0.1M NaOH, and 3.5% NaCl for 5 min, 10 min, and 12 h, respectively, and the surfaces still exhibited water repellency well as shown in Figure S5 (Supporting Information), indicating that the coating film had excellent stability for the practical application.

### 3.6. Fabrication of Mini-Foam-Copper Boat and As-Spun PS Fiber

A sheet of copper foam was folded to a miniature boat with 4 cm × 2 cm × 1 cm. Then, the miniature ship was successively washed with deionized water and ethanol to remove surface impurities. Afterward, the superhydrophobic mini-boat was fabricated according to the above method: Etched in 10 mg mL^−1^ FeCl_3_ and 0.1 M HCl solution with ultrosonication about 10 min, followed by immersion in an ethanol solution of ODE (1 mM) for 10 min [32]. The as-prepared miniature boat floated freely on the water surface when in contact with water. The miniature boat weighs 1.2 g itself, but with a maximum loading weight of 12 g. Moreover, the folded boat has a height of 1 cm, but can endure a water pressure of 10 cm height.

The combination of superhydrophobic foam Cu boat and electrospun fiber with high oil absorption capacity may provide a good solution for the convenient collection of oil spill. Herein, one-step electrospinning to produce PS fibers with high oil adsorption capability was carried out by changing the compositions of polymer solutions; the PS fibers with different porous morphology and adsorption-capacity were tailored effectively [45]. In our experiments, the concentration of PS (300 mg mL^−1^) and DMSO/CB with 30/70 (*v*/*v*) was kept constant. The surfactants of CTAB, SDS, Tween 80, and Trition-X-100 were added with a critical micelle concentration (CMC) and 1% (*w*/*v*), respectively. The identical experiments with or without 1% concentration of TBAP were carried out. The formulation, the as-spun fiber diameters, and their oil absorption capacity are listed in Table 2. Due to the low dielectric constant, chlorobenzene (CB) alone cannot be used to produce dry PS fibers, the different surfactants, such as CTAB, SDS, Tween 80, and Trition-X-100, and TBAP as an organic salt are commonly added to enhance the conductivity of the solutions. The addition of DMSO as a high dielectric constant solvent can play an important role in the formation of the pores. These additives could enhance the electrospinning ability of the solution and produce bead-free fibers. Moreover, they might change critical parameters for electrospinning, such as the solvent quality to polymers, the viscosity, and the surface tension of solutions. As shown in Table 2, the WCAs of as-spun fibers prepared using different additives were in the range of (124.2 ± 4.2)°–(132 ± 2.2)°, with a fiber diameter of 1.6–3.9 μm. The roughness was created by the combination of the voids between fibers and the microscale pores, leading to a hydrophobic surface. The oil adsorption capacity for each fiber was investigated with gasoline engine oil with the largest absorption capacity being more than 85 g g^−1^ (b in Table 2) using the selected condition [45,46].

**Figure 7:**
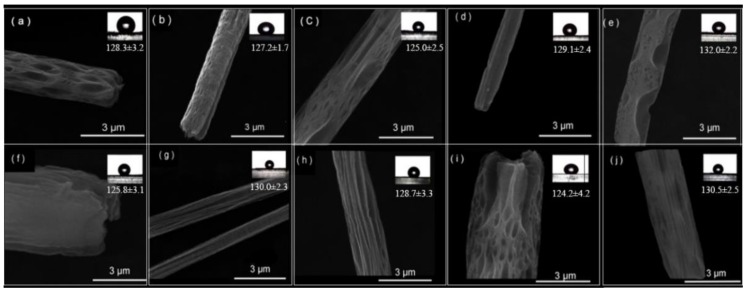
SEM micrographs of the surfaces (**a**–**j**) and the WCAs of the as-spun PS fibers fabricated from solutions with varying compositions.

According to the SEM images (Figure 7), the diameter of the PS fibers is uniform, and, more importantly, the nonsolvent induces a highly porous structure throughout the fibers during the drying process. By combing the polar nonsolvent with the organic salt or surfactants, the as-spun fibers with small diameters and highly porous structures possess an exceptional ability to adsorb oils. Voids among fibers were the key for the high oil absorption capacity.

**Figure 8:**
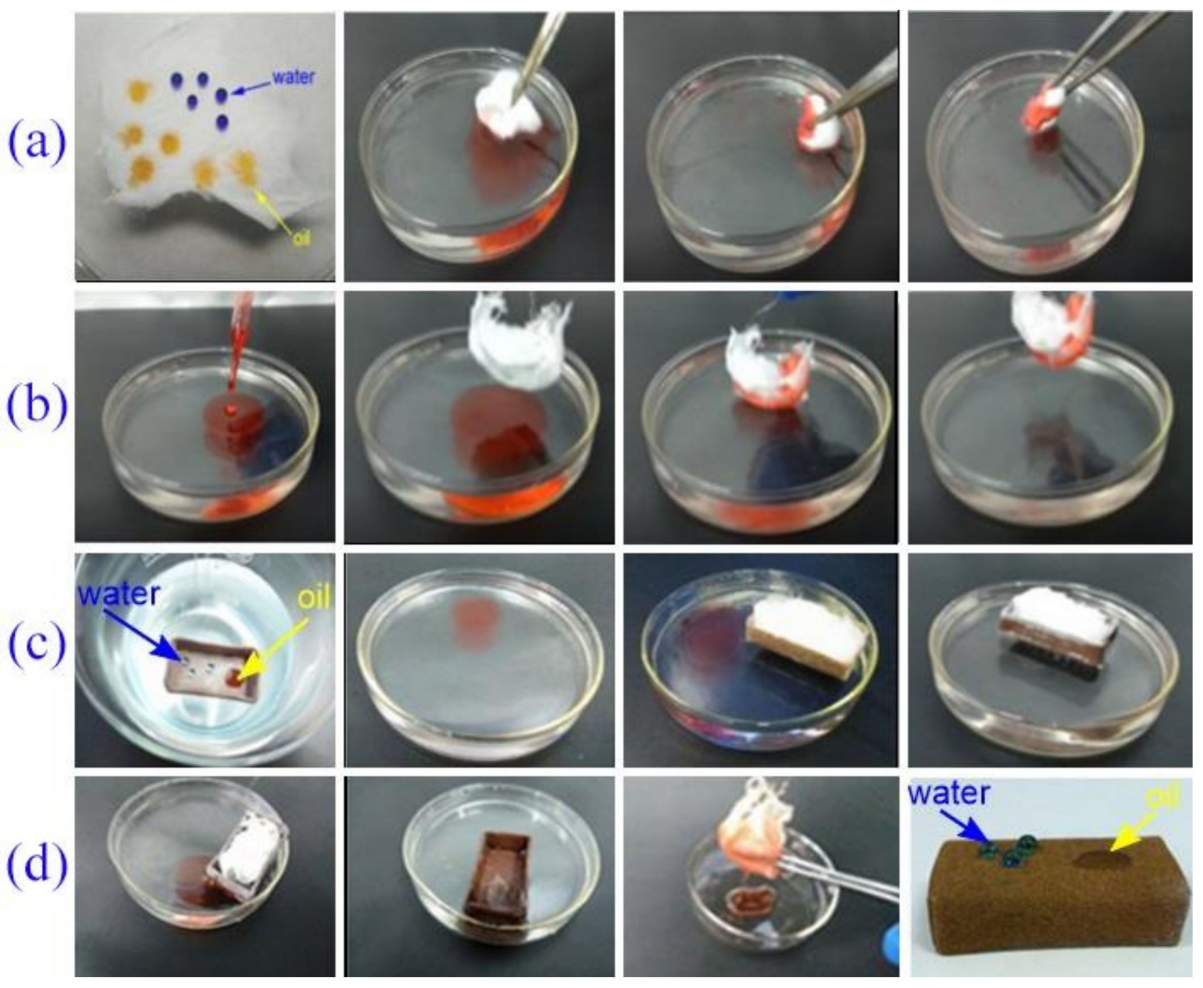
Oil absorption and collection using the as-spun PS fibers (**a**), the fibers embedded with a string bag (**b**), the superhydrophobic mini-boat (**c**), and the mini-boat loaded with the PS fibers (**d**), respectively.

The single and combinational modes using the mini-boat modified with ODE and as-spun fiber with the highest oil absorption capacity (b in Table 2) were conveniently applied for the collection of oil spill, respectively. As presented in Figure 8a, the water droplets dyed with methylene blue performed like round balls whilst the red oil droplets dyed with Sudan red dispersed on the as-spun fiber surfaces. Additionally, similar phenomena were found in the mini-boat inner surface with water droplets sitting like a ball and oil droplets scattering on the floor of the mini-boat (the first picture in Figure 8c). The as-spun PS fibers, the fibers embedded with a string bag, the superhydrophobic mini-boat, and the boat loaded with PS fibers were immersed in the oil over the water surface in the Petri dish, respectively, and effective oil/water separation were realized visually as shown in Figure 8. After over 10 times oil absorption and collection, the superhydrophobic and oleophilic properties still remained for the mini-boat. It could be seen that bulbiform water droplets could sit on the surface of the upside mini-boat, but oil droplets dispersed and penetrated its surface (the last picture in Figure 8d). In view of the superhydrophobic and oleophilic properties for both materials, high oil absorption capacity for the PS fibers, and rapid oil release by squeezing, these flexible approaches could conveniently apply to oil-water separation.

## 4. Conclusions

In summary, superhydrophobic copper foam was rapidly fabricated by two simple steps and the optimal conditions were achieved by using 10 mg mL^−1^ FeCl_3_/HCl and 10 mM ODE ethanol solution, respectively. Likewise, the designed mini-boat exhibited excellent superhydrophobicity and superoleophilicity with a load about 10 times of its weight. Moreover, the as-spun PS fibers were produced with the highest oil absorption capacity of 85 times of its weight when using 1% (*w*/*v*) CTAB as an additive during electrospinning. Both the as-prepared superhydrophobic mini-ship and the as-spun hydrophobic PS fiber could separate and collect the oil over the water surface in the single or combinational mode and the adsorbed oil could be collected by simple squeezing. The simplicity, fabrication speed, and low cost make this approach potentially suited to industrialization, but further studies about the design of the solid filtration set-up, scale-up experiments, and hydrophobic recovery after long-term use should be done in order to be applied for large scales of oil/water separation.

## Figures and Tables

**Table 1:** The concentration optimization of ODE for the WCA and absorbing amount of engine oil.

C (mM)	1	5	10	15	20
WCA (°)	154.4 ± 1.6	158.3 ± 1.5	154 ± 1.7	150 ± 1.5	156 ± 1.7
m_1_ (g)	0.0447	0.0443	0.0416	0.0417	0.0403
m_2_ (g)	0.4638	0.1967	0.1981	0.1957	0.1856
*A*_c_	3.5	3.4	3.8	3.7	3.6

**Table 2:** Relationship of solution compositions and the characteristics of as-spun fibers.

No.	CTAB (*w*/*v* %)	SDS (*w*/*v* %)	Tween80 (*w*/*v* %)	Trition-X-100 (*w*/*v* %)	TBAP (*w*/*v* %)	Fiber Diameter (μm)	Absorption Capacity (g g^−1^)
a	0.032	-	-	-	-	3.4 ± 0.1	60.2 ± 4.3
b	1.0	-	-	-	-	2.2 ± 0.5	85.4 ± 3.2
c	-	0.032	-	-	-	2.7 ± 0.1	51.2 ± 4.4
d	-	1.0	-	-		1.5 ± 0.3	72.8 ± 2.5
e	-	-	0.55	-	-	2.8 ± 0.2	42.2 ± 4.6
f	-	-	1.0	-	-	3.9 ± 0.1	40.5 ± 5.1
g	-	-	-	0.012	-	1.6 ± 0.6	43.1 ± 3.5
h	-	-	-	1.0	-	2.1 ± 0.2	44.5 ± 2.7
i	-	-	-	-	0.0	3.7 ± 0.1	24.4 ± 5.3
j	-	-	-	-	1.0	2.4 ± 0.2	34.8 ± 6.4

## Supplementary Materials

The following are available online at http://www.mdpi.com/2073-4360/11/1/97/s1.
