# Supplementary material for: Facile Fabrication of Superhydrophobic Copper-Foam and Electrospinning Polystyrene Fiber for Combinational Oil–Water Separation
Source: Polymers (Basel). 2019 Jan 8;11(1):97. doi: 10.3390/polym11010097 (PMC6402002; doi:10.3390/polym11010097)
Supplement: Supplementary file 1 [file polymers-11-00097-s001.pdf]

Supporting Information

Facile Fabrication of Superhydrophobic Copper-foam and Electrospinning Polystyrene Fiber for Combinational Oil–Water Separation

Yu-Ping Zhang [1,*], Jing-Hua Yang [1,2], Ling-Li Li [1], Cheng-Xing Cui [1], Ying Li [1], Shan-Qin Liu [1], Xiao-Mao Zhou [2,*] and Ling-Bo Qu [3]

[1] Henan Institute of Science and Technology, Xinxiang 453000, China; (Y. -P. Z.); (J. -H. Y.); (L. -L. L.); (C. -X. C.); (Y. L.); (S. -Q. L.) chengxingcui@hist.edu.cn
[2] Hunan Academy of Agricultural Sciences, Changsha 410128, China; (X.-M. Z.)
[3] College of Chemistry and Molecular Engineering, Zhengzhou University, Henan 450001, China; (L. B. Q.) qulingbo@zzu.edu.cn
* Correspondence: zhangyuping@hist.edu.cn (Y. -P. Z.); beijing2017xmz@163.com (X. -M. Z.)

Scheme S1. Illustration for the fabrication process of the superhydrophobic copper foams using 4 sulfhydryl compounds.

Figure S1. The images of etched copper foams after water washing in the presence of nitrogen blowing for drying (a) and absence of nitrogen blowing (b-d, after stay in 5-6 h). The etched process was carried out using 0.01g/ml $FeCl_3$ about 15min.

Table S1. The absorption capacity of gasline engine oil using different concentration of $FeCl_3$.

Figure S2. The Optimization of $FeCl_3$ (mg/mL) concentration in the chemical etching process.

Figure S3. SEM characterization of copper foams after modification by ODE ethanol solution with a different time of 5,10,15,20 and 25 min, respectively.

Figure S4. Change of absorption amount for CCl_4 using the as-prepared copper foam during 10 cycles.

Figure S5. Upper: SEM characterization of foam copper after modification of ODE immersed

about 12h in some rigid conditions. Below: The changes of WCAs in different rigid

conditions.

Video 1: Superhydrophobicity of Cu foams modified by EE, DE, HDE and ODE.

Video 2: Determination of the WSA with a sliding angle of 0°.

Video 3: Determination of the WSA with a sliding angle of 5°.

Video 4: Determination of the WSA with a sliding angle of 10°.

Video 5: Test to absorb the oil.

Video 6: Oil–water separation.

Supplementary files

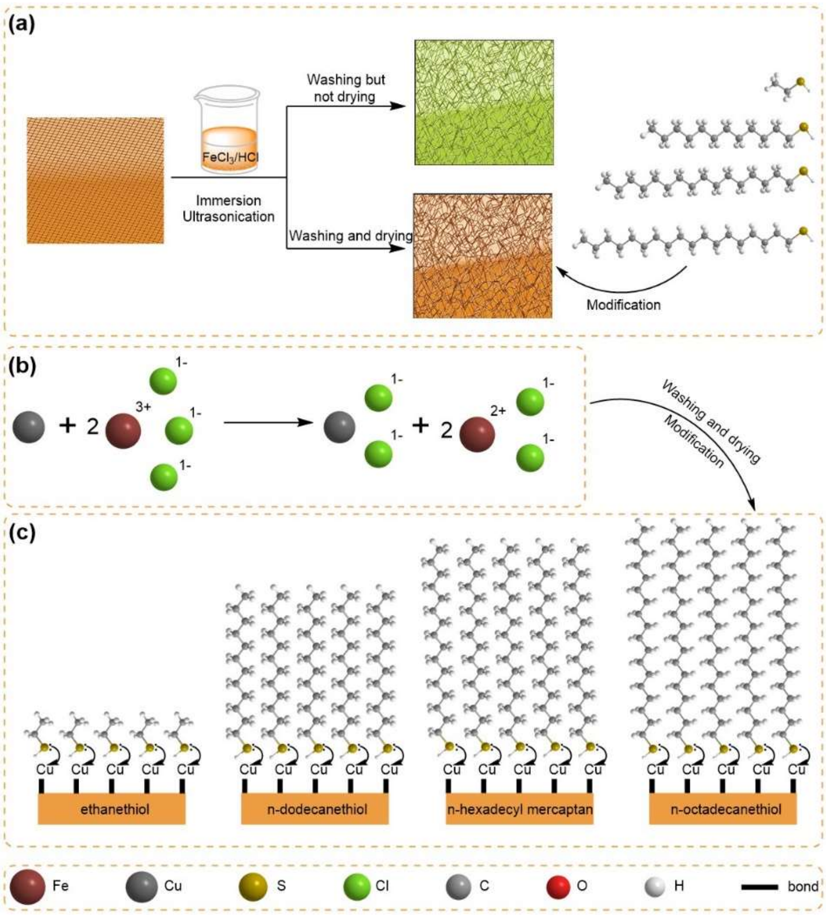

Scheme S1. Illustration for the fabrication process of the superhydrophobic copper foams using 4 sulfhydryl compounds.

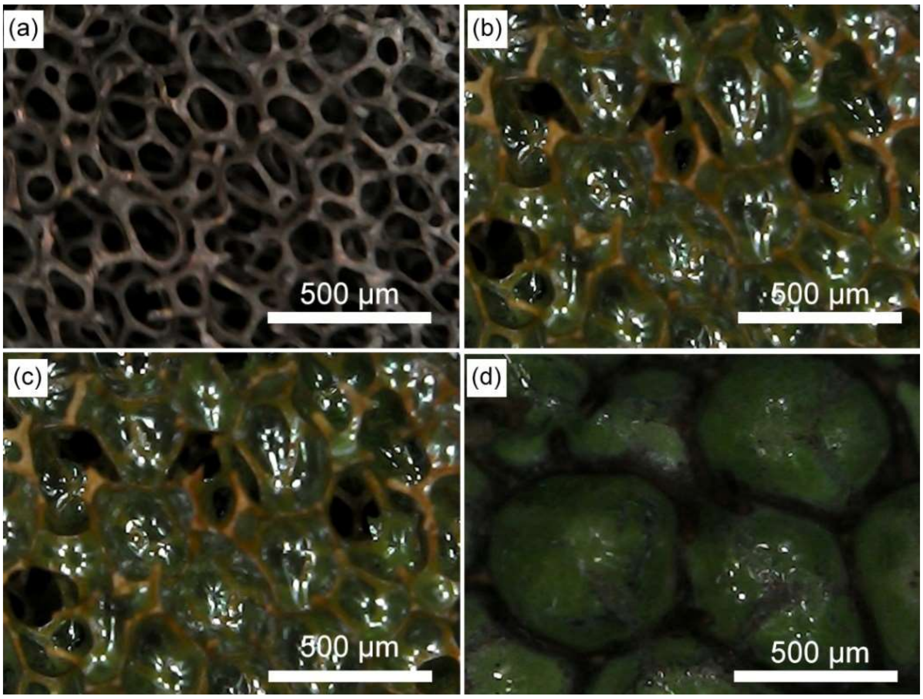

Figure S1. The images of etched Cu foams after water washing in the presence of nitrogen blowing for drying (a) and absence of nitrogen blowing (b-d, after stay in 5-6 h). The etched process was carried out using 0.01g/ml $FeCl_3$ about 15min.

Table S1. The absorption capacity of gasline engine oil using different concentration of $FeCl_3$.

C (mg /mL)	2.5	5	10	20	40
m_1 (mg)	57.2	47.1	31.9	5.6	0
m_2 (mg)	275.9	227.5	201.8	/	/
A_C	3.8	3.8	5.3	/	/

The absorption capacity is calculated according to $A_c = (m_2 / m_1) \times 100\%$, where m_1 and m_2 were the Cu foam mass before and after absorbing the oil, respectively.

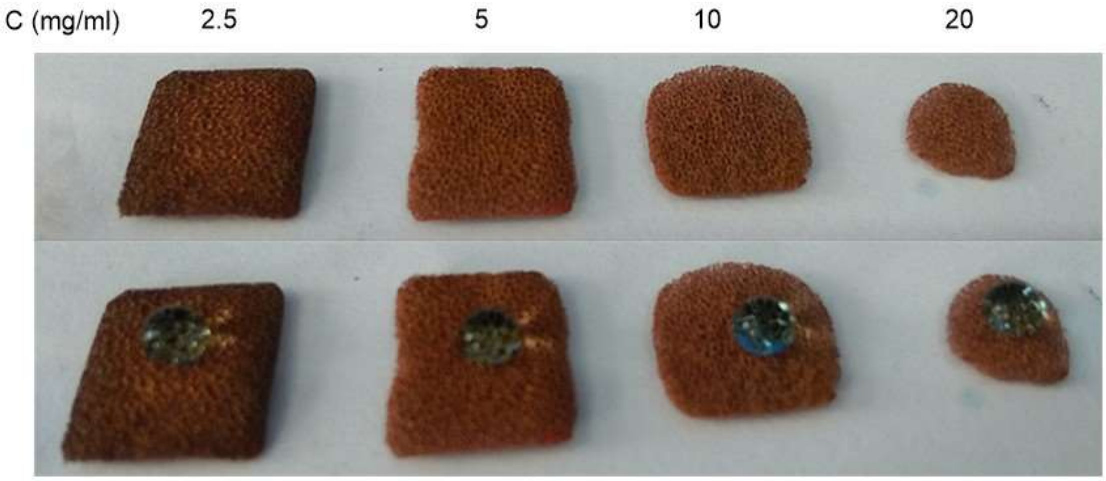

Figure S2. The Optimization of $FeCl_3$ concentration in the chemical etching process.

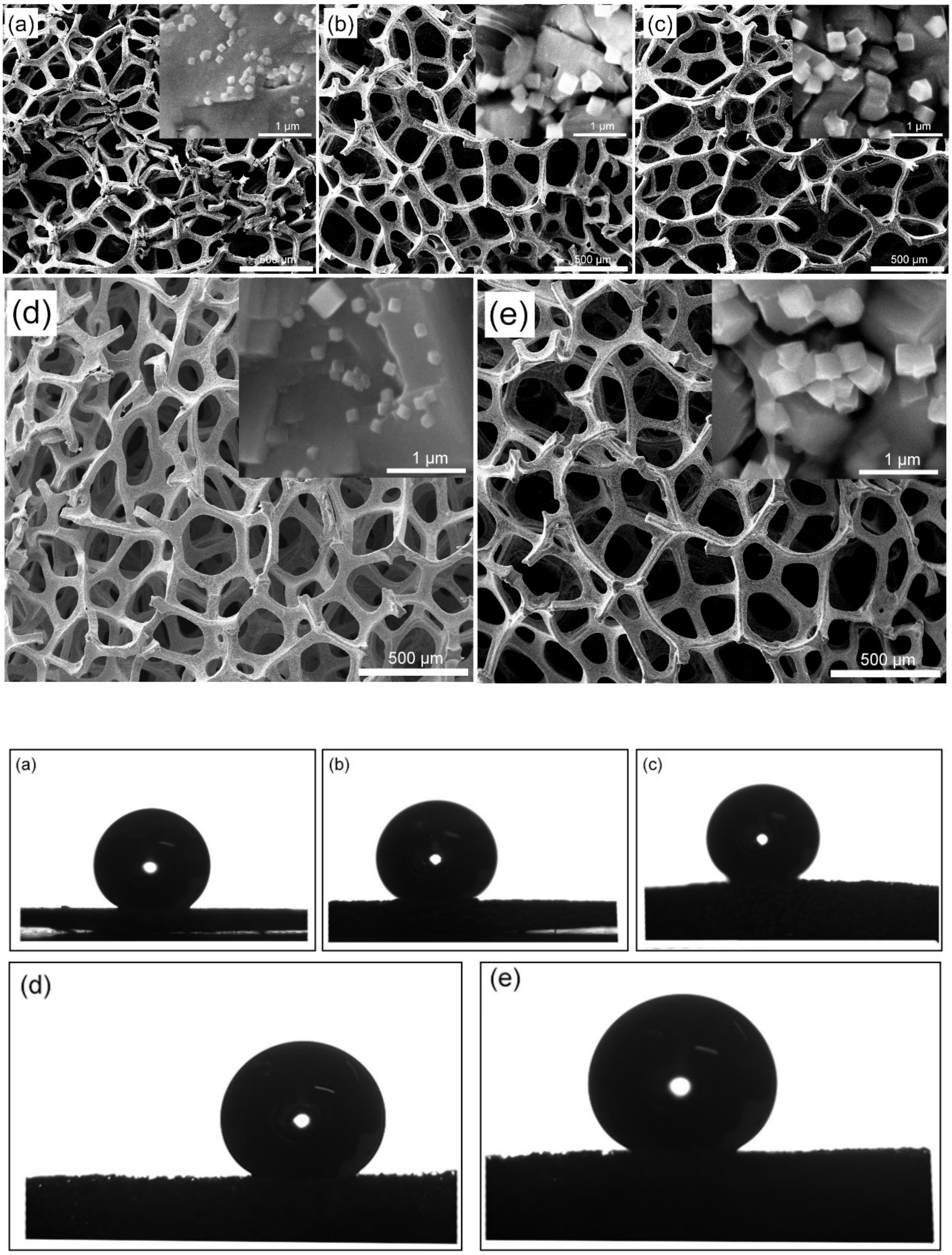

Figure S3. SEM characterization of copper foams after modification by 1 mM ODE ethanol solution with a different time of 5,10,15,20 and 25 min.

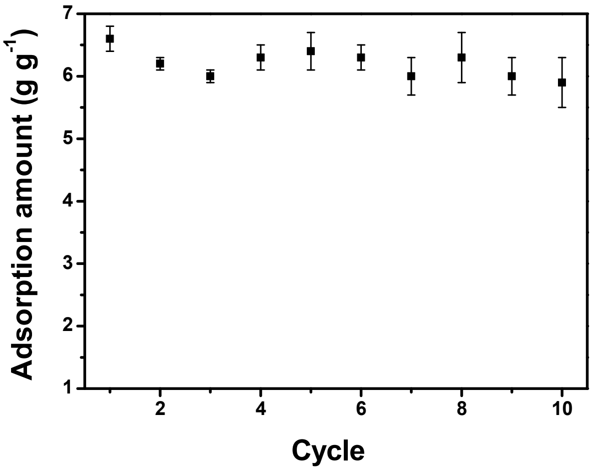

Figure S4. Change of absorption amount for CCl_4 using the as-prepared copper foam during 10 cycles.

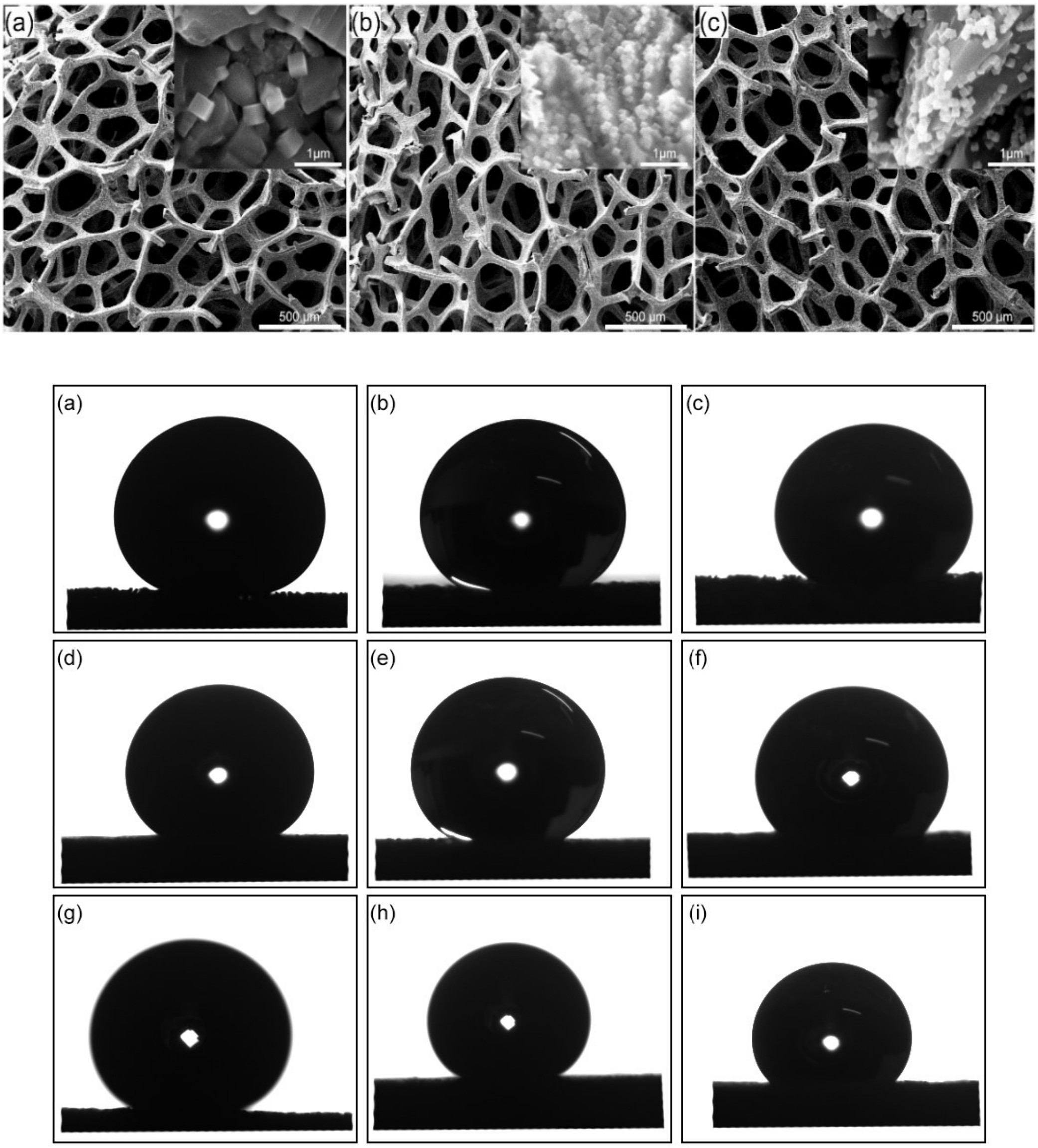

Figure S5. Upper：SEM characterization of foam copper after modification of ODE immersed about 12h in some rigid conditions. (a)-(c) stand for the condition of pH=1, pH=13 and 3.5% NaCl, respectively. Below: The changes of WCAs in different rigid conditions, (a)-(c) stand for the WCAs after the porous Cu is immersed about 5 min, 10 min and 12h, respectively, in a solution with a pH of 1.（d）-（f）stand for the WCAs after the porous Cu is immersed about 5 min, 10 min and 12h, respectively, in a solution with a pH of 13. （g）-（i） stand for the WCAs after the porous Cu is immersed about 5 min, 10 min and 12h, respectively, in a solution with a 3.5% NaCl.
